# Performance of the MRI lesion pattern score in predicting neurological outcome after out of hospital cardiac arrest: a retrospective cohort analysis

**DOI:** 10.1186/s13054-024-05007-w

**Published:** 2024-07-02

**Authors:** Manuela Iten, Antonia Moser, Franca Wagner, Matthias Haenggi

**Affiliations:** 1 Department of Intensive Care Medicine, Inselspital, University Hospital Bern, Bern, Switzerland; 2 University Institute for Diagnostic and Interventional Neuroradiology, Inselspital, University of Bern, Bern, Switzerland; 3 Institute of Intensive Care Medicine, University Hospital Zurich, Zurich, Switzerland

**Keywords:** Cardiac arrest, Neuroprognostication, MRI, MR lesion pattern, MRI score, Out of hospital cardiac arrest, OHCA

## Abstract

**Background:**

Despite advances in resuscitation practice, patient survival following cardiac arrest remains poor. The utilization of MRI in neurological outcome prognostication post-cardiac arrest is growing and various classifications has been proposed; however a consensus has yet to be established. MRI, though valuable, is resource-intensive, time-consuming, costly, and not universally available. This study aims to validate a MRI lesion pattern score in a cohort of out of hospital cardiac arrest patients at a tertiary referral hospital in Switzerland.

**Methods:**

This cohort study spanned twelve months from February 2021 to January 2022, encompassing all unconscious patients aged ≥ 18 years who experienced out-of-hospital cardiac arrest of any cause and were admitted to the intensive care unit (ICU) at Inselspital, University Hospital Bern, Switzerland. We included patients who underwent the neuroprognostication process, assessing the performance and validation of a MRI scoring system.

**Results:**

Over the twelve-month period, 137 patients were admitted to the ICU, with 52 entering the neuroprognostication process and 47 undergoing MRI analysis. Among the 35 MRIs indicating severe hypoxic brain injury, 33 patients (94%) experienced an unfavourable outcome (UO), while ten (83%) of the twelve patients with no or minimal MRI lesions had a favourable outcome. This yielded a sensitivity of 0.94 and specificity of 0.83 for predicting UO with the proposed MRI scoring system. The positive and negative likelihood ratios were 5.53 and 0.07, respectively, resulting in an accuracy of 91.49%.

**Conclusion:**

We demonstrated the effectiveness of the MLP scoring scheme in predicting neurological outcome in patients following cardiac arrest. However, to ensure a comprehensive neuroprognostication, MRI results need to be combined with other assessments. While neuroimaging is a promising objective tool for neuroprognostication, given the absence of sedation-related confounders—compared to electroencephalogram (EEG) and clinical examination—the current lack of a validated scoring system necessitates further studies. Incorporating standardized MRI techniques and grading systems is crucial for advancing the reliability of neuroimaging for neuroprognostication.

*Trial Registration*: Registry of all Projects in Switzerland (RAPS) 2020-01761.

## Background

Despite advancements in resuscitation practice, sudden cardiac death ranks as third leading cause of death in Europe, and less than 10% of patients experiencing out-of-hospital cardiac arrest (OHCA) achieve a favourable outcome (FO) [1]. The health and economic burden stemming from cardiac arrest is substantial [2, 3]. Following the resolution of initial hemodynamic instability, the primary complication of cardiac arrest is neurological dysfunction arising from ischemic/hypoxic brain injury during the event and reperfusion injury post-successful resuscitation [4, 5]. Minimizing the period of uncertainty for the patient’s family, avoiding unnecessary treatments and consequently reducing costs underscore the critical need for a prompt and accurate neurological prognostication.

Unfortunately, early and precise assessment of the severity of the sequelae and the neurological impact remains challenging [6, 7]. Achieving high predictive accuracy in neuroprognostication after cardiac arrest requires a multimodal approach. This approach integrates clinical signs of coma with electroencephalography (EEG), biochemical parameters (NSE, neuron-specific enolase), radiological imaging (MRI, CT), or somatosensory evoked potentials (SSEP) [7, 8]. While the majority of signs and examinations have proven ineffective in predicting outcomes in larger trials, combining them has demonstrated significant power for predicting unfavourable outcome (UO) at 72 h [8]. However, the specificity of multimodal prognostication remains low, resulting in a considerable number of patients receiving an ‘indeterminate’ prognosis. Although combinations of biomarkers may offer assistance for prediction of FO [9], they have yet to be integrated into clinical practice. Criteria such as continuous and reactive EEG, a GCS motor score above 3, SSEP N20 wave amplitude > 4 µV, normal NSE levels, and normal MRI findings suggest a FO, albeit with a sensitivity of approximately 40% [6]. Combining these predictors, including brain imaging with either early CT scans conducted on days 2–3 or MRI diffusion weight imaging (DWI) after approximately 80 h, classified as absent, mild, or extensive hypoxic brain injury, has demonstrated reasonable sensitivity and specificity [10]. While many of the suggested examinations can be quantified or categorized, the interpretation of brain imaging depends on subjective evaluations [10].

To diminish the duration of prognostic uncertainty, numerous patients in the Intensive Care Unit (ICU) at the Inselspital University Hospital Bern, Switzerland undergo early cerebral MRI (within 24 h up to 72 h) following cardiac arrest. We introduced a classification scheme designed to characterize the ischemic burden subsequent to hypoxic-ischemic encephalopathy [13]. However, the validity of this classification scheme remains uncertain.

This study endeavours to elucidate the performance and validity of the proposed classification scheme in a cohort of OHCA patients.

## Methods

This retrospective cohort study was performed at the Department of Intensive Care Medicine, Inselspital, University Hospital Bern, Switzerland. Data were retrospectively analysed from the Bern resuscitation registry, a prospective collection of data (following to the Utstein style) for all OHCA patients. The registry is approved by the cantonal ethics committee of the Canton Bern, Switzerland (Project-ID 2020-01761). Patients or their next of kin were informed and consented to the use of the collected data, with those facing language barrier excluded from the participation. This report adheres to the applicable reporting of studies conducted using observational routinely collected data (RECORD) guidelines.

We screened all patients admitted to the adult ICU at Inselspital, University Hospital Bern, Switzerland between February 1, 2021 and January 31, 2022, who were ≥ 18 years old, unconscious, intubated and not following commands (defined as FOUR score motor response ≤ 3) after cardiac arrest and had sustained ROSC. Treatment involved 24 h normothermia (≤ 36 °C) followed by fever avoidance for a total of 72 h after ROSC, in accordance with local protocol and current guidelines. If a patient remained unconscious (defined as not being able to follow commands, FOUR score motor response ≤ 3) after 24 h of normothermia and sedation hold, ancillary testing (EEG, NSE and brain imaging) was conducted, following guidelines [11, 14], to facilitate neuroprognostication at the earliest possible time point beyond 72 h post ROSC. Neurological outcome was assessed by phone calls at days 30 and 180 after cardiac arrest, graded with the cerebral performance categories (CPC) scale (ranging from 1—good neurological performance to 5—brain death). Best CPC during this time period was used for analysis to prevent misclassification of patients who recovered and subsequently died for unrelated reasons of the index hospitalisation [15].

**Fig. 1:**
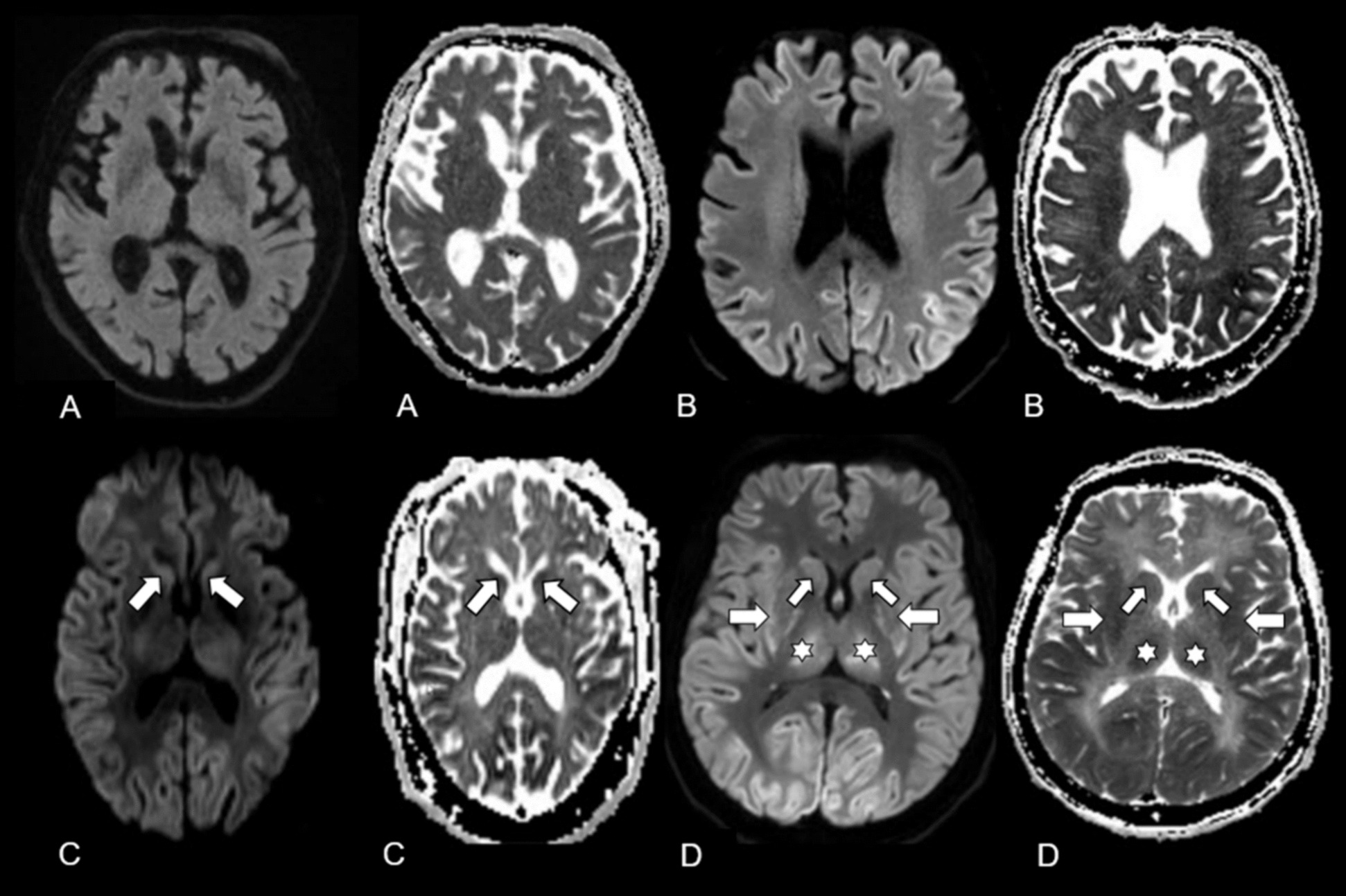
Representative examples for the four MRI lesion pattern (MLPs)

MRI acquisition was routinely performed with 3 T Siemens MR Scanners (Magnetom Vida, Magnetom Verio or Magnetom Skyra fit; Erlangen/Germany). Certified staff neuro-radiologists from the Department of Neuroradiology of the Inselspital, University Hospital Bern quantified MRI findings as part of their clinical routine. The MRI report based on four different patterns known as MR-lesion patterns (MLP), originally described by Barth et al. [13]. According to this publication, MRI findings were classified based on the DWI and apparent diffusion coefficient ADC restrictions. An axial T2w and a coronal T2w-FLAIR (Fluid-Attenuated Inversion Recovery) were used to detect old hyperintense abnormal signal alterations, to exclude chronic infarction, or as a reference to exclude T2- “shine through” effect. ADC values were measured in pre-defined regions of interest located in the cerebral cortex, the cerebellar cortex, the hippocampi, the basal ganglia, both thalami and the brain stem. The cerebral cortex contained eight regions of interest’s (one in each frontal, parietal, temporal, and occipital lobe). Region of interest sizes were 4 mm^2^ for the cerebral and cerebellar cortex and the hippocampi, 10 mm^2^ for the basal ganglia, thalami and the brain stem. Regions of interests that revealed restricted diffusion and corresponding decreased ADC values < 650 × 10^–6^ mm^2^ were considered as pathologically restricted as previously suggested [16]. Barth and colleagues then defined four different patterns called MR-lesion patterns (MLPs) based on DWI/ADC restriction in the different regions of interest. MLP 1 was defined as an absence of any gray matter lesion; MLP 2 as purely cortical grey matter lesions; MLP 3 as the presence of basal ganglia lesions without involvement of other subcortical grey matter (with or without cortical lesions); and MLP 4 as lesions of the thalami and/or hippocampi and/or brain stem (with or without cortical or basal ganglia lesion). Representative examples are displayed in Fig. 1.

Neuroradiologists were blinded to the patients’ outcome. Outcome assessors where blinded to the MRI results and additional exams.

For each subject the diffusion weighted imaging (DWI, left) and apparent diffusion coefficient (ADC, right) are presented. (A) MLP 1: normal brain MR scan of a 82-year-old male with absence of grey matter lesion. (B) MLP 2: MR scan of a 77-year-old male with symmetric involvement of the frontal and parietal cortex in absence of subcortical lesions. (C) MLP 3: MR scan of a 74-year-old male with involvement of the basal ganglia (arrows) and of the cortex (fronto-temporo-parieto-occipital bi-hemispherical symmetric). (D) MLP 4: MR scan of a 75-year-old female with involvement of the thalami (stars), the basal ganglia (arrows) and the fronto-temporo-parieto-occipital cortex bi-hemispherical symmetric. [Originally published by Barth et al. [13], with permission from Elsevier].

### Statistical analysis

Data are presented as median and interquartile range [IQR]. The outcome was dichotomized into favourable (CPC 1 and 2) and unfavourable (CPC 3–5), MRI-lesion patterns were dichotomized into severe hypoxic brain injury (MLP 3–4) and no or minimal hypoxic brain injury (MLP 1–2). Measures of diagnostic accuracy (sensitivity, specificity, positive predictive value, negative predictive value, positive likelihood ratio, negative likelihood ration and accuracy) were calculated accordingly.

## Results

**Fig. 2:**
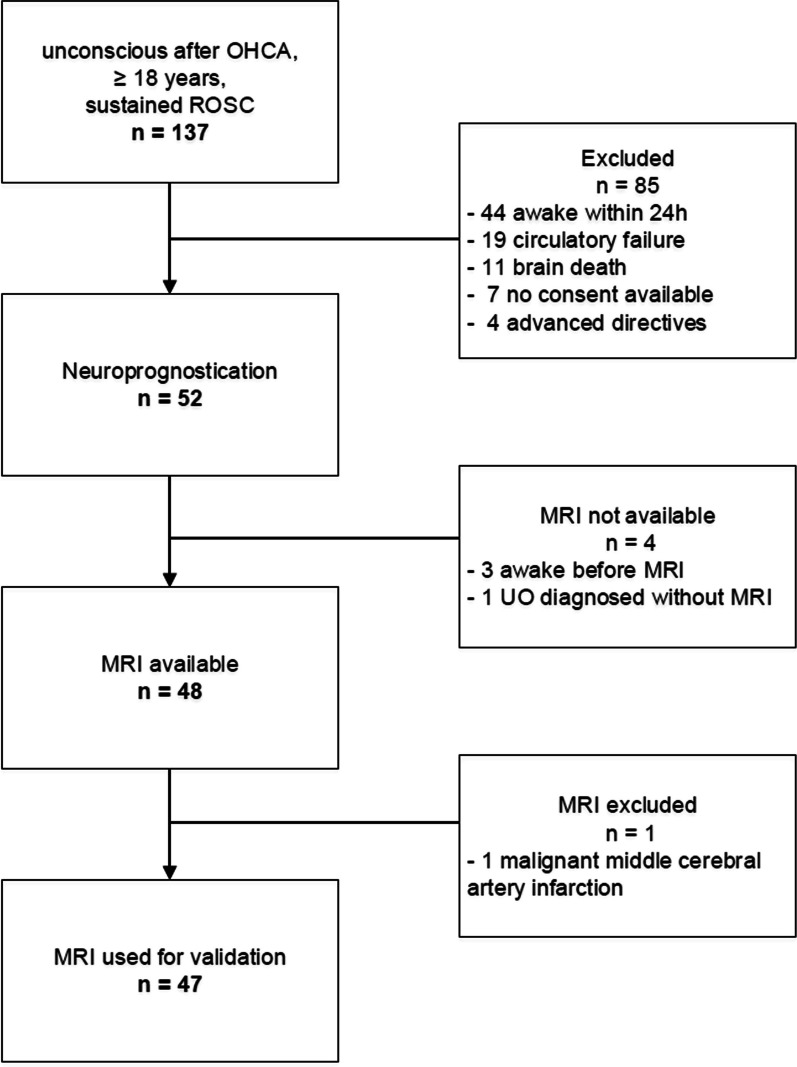
Consort diagram. *OHCA* out of hospital cardiac arrest, *ROSC* return of spontaneous circulation, *UO* unfavourable outcome

Among the 137 patients admitted to the ICU with OHCA from February 1, 2021 to January 31, 2022, 52 (38%) patients entered the process of neuroprognostication (Fig. 2). Excluded from the study were 85 patients: 44 regained consciousness within the first 24 h, 19 succumbed to circulatory failure, eleven were brain death, seven did not consent for the registry, and four patients had explicitly stated advanced directives that were not fully aligned with comprehensive ICU care.

### Baseline data

The median age was 66 years [50–74] with 83% being male (n = 43). Patients with UO were older (69 years, [60–75]) and significantly more often male (86%) (Table 1). Baseline cardiac arrest data, such as witnessed arrest, initial rhythm, no flow and low flow time did not differ between UO and FO groups. Coronary angiography and percutaneous transluminal coronary angioplasty (PTCA) were equally distributed in both groups. Regarding of neuroprognostication, more patients in the UO group underwent EEG and had NSE tested. All patients (100%) had a clinical neurological examination and 92% of all patients entering the process of neuroprognostication received an MRI (Table 1).

**Table 1:** Demographic data

	All Patients		UO		FO		
	(n = 52)		(n = 37)		(n = 15)		*p* value
	n	%	n	%	n	%	
Male	**43**	83	**32**	86	**11**	73	< 0.0001
Female	**9**	17	**5**	14	**4**	27	
Age (years) [mean—IQR]	**66**	50.5–74	**69**	60–75	**53**	46–65	0.0187
*Medical history*							
Arterial hypertension	**16**	31	**14**	38	**2**	13	
Coronary heart disease	**30**	58	**20**	54	**10**	67	
Diabetes mellitus	**5**	10	**4**	11	**1**	7	
*Details cardiac arrest*							
Witnessed arrest	**39**	75	**26**	70	**13**	87	0.2438
Unknown	**4**	8	**3**	8	**1**	7	
Defibrillation	**37**	71	**26**	70	**11**	73	
No flow (min) [mean—IQR]	**1**	0–5	**2.5**	0–5	**1**	0–2	0.714
Low flow (min) [mean—IQR]	**25**	20–38	**27.5**	20–38	**20**	12–30	0.5467
Initial rhythm shockable	**30**	58	**19**	51	**11**	73	0.2037
Initial rhythm non-shockable	**17**	33	**14**	38	**3**	20	
Initial rhythm unknown	**5**	10	**4**	11	**1**	7	
*Interventions*							
Coronary angiography	**32**	62	**21**	57	**11**	73	0.2523
PTCA	**20**	38	**12**	32	**8**	53	0.2189
ECMO	**2**	4	**1**	3	**1**	7	
Impella	**1**	2	**0**	0	**1**	7	
*Causes of cardiac arrest*							
Myocardial infarction	**20**	38	**12**	32	**8**	53	
Arrhythmia	**9**	17	**7**	19	**2**	13	
Hypoxia	**5**	10	**3**	8	**2**	13	
Hypovolaemia	**3**	6	**2**	5	**1**	7	
Intoxication	**2**	4	**2**	5	**0**	0	
Hyperkalaemia	**1**	2	**1**	3	**0**	0	
Pulmonary Embolism	**0**	0	**0**	0	**0**	0	
Trauma	**0**	0	**0**	0	**0**	0	
Unknown	**12**	23	**10**	27	**2**	13	
*Neuroprognostication*							
Complete neuroprognostication	**51**	98	**36**^a^	97	**15**	100	
Clinical exam	**52**	100	**37**	100	**15**	100	
≥ 1 EEG	**47**	90	**34**	92	**13**	87	
≥ 1 NSE	**51**	98	**37**	100	**14**	93	
SSEP	**0**	0	**0**	0	**0**	0	
MRI	**48**	92	**36**	97	**12**	80	
Time from ROSC to MRI (h) [mean—IQR]	**54**	48–72	**54**	48–70	**53**	48–93	

Bold are the numbers (n), normal the percentage

Data presented as number and percentage, respectively mean and interquartile range IQR. *UO* unfavourable outcome, *FO* favourable outcome, *PTCA* percutaneous transluminal coronary angioplasty, *ECMO* extra-corporal membrane oxygenation, *EEG* electroencephalogram, *NSE* neuron specific enolase, *SSEP* somatosensory evoked potential, *ROSC* return of spontaneous circulation

^a^One patient with malignant middle cerebral artery infarction did not have completed neuroprognostication

### Outcome data

Among the 52 patients undergoing neuroprognostication, three patients regained consciousness bevor MRI was performed and one patient was diagnosed with UO without MRI since he had no pupillary and corneal reflexes, EEG was highly malignant and NSE was elevated twice, following current guidelines [8] (Fig. 2). This resulted in a cohort of 48 patients with available MRI data. One patient diagnosed with malignant middle cerebral artery infarction in the MRI was excluded from the validation process (Fig. 2).

Of the remaining 47 patients with MRIs, 35 exhibited signs of severe hypoxic brain injury (MLP 3–4), with 33 of them (94%) experiencing UO, while 2 had a FO (6%) (Table 2). In only 5 out of 35 patients with UO, MRI was one of the two additional modalities predicting UO. All of these 5 patients hat an MLP score of 4 and an elevated NSE, additionally one presented with possible status myoclonus, one with a malignant EEG pattern, one with moderate to extended hypoxic brain injury on CT and one with an absent pupillary but still present corneal reflex. Among the twelve patients with minimal or no lesion (MLP 1–2), ten (83%) had FO, and two (17%) had UO (Table 2). Both patients with UO had no lesions on MRI (Table 2), consistent with other neuroprognostication exams: a GCS motor score of 5 at 72 h after ROSC, NSE repeatedly below the threshold, EEG without highly malignant patterns, and present pupillary and corneal reflexes. One patient had clearly stated advanced directives and died 10 days after ROSC with a maximum GCS of 10. The other patient succumbed on day 26 with a maximum GCS of 7.The sensitivity of an MLP 3–4 to predict UO was 0.94, with a specificity of 0.83, resulting in a positive predictive value of 0.94 and a negative predictive value of 0.83 (Table 3). The positive likelihood ratio and negative likelihood ratio were 5.53 and 0.07, respectively, with an overall accuracy of 91.49% (Table 3).

**Table 2:** Distribution of MR lesion patterns across unfavourable and favourable outcome

	MLP 4	MLP 3	MLP 2	MLP 1	Total
Unfavourable Outcome (CPC 3–5)	29	4	0	2	35
Favourable Outcome (CPC 1–2)	2	0	2	8	12
Total	31	4	2	10	47

1 MRI excluded with infarction of the middle cerebral artery

**Table 3:** Diagnostic performance parameters

					CI
	UO	FO	Sensitivity	0.94	0.79 –0.99
	(CPC 3–5)	(CPC 1–2)	Specificity	0.83	0.51 –0.97
MLP 3–4	33	2	Positive predictive value	0.94	0.97 –0.99
			Negative predictive value	0.83	0.51 –0.97
MLP 1–2	2	10	Positive likelihood ratio	5.53	1.59 –20.01
			Negative likelihood ratio	0.07	0.02 –0.27
			Accuracy (%)	91.49	
			False Positive Rate	0.057	0.01 –0.21
			False Negative Rate	0.167	0.03 –0.49

*MLP* MR lesion patterns, *UO* unfavourable outcome, *FO* favourable outcome, *CPC* cerebral performance category

## Discussion

In our cohort study, we demonstrated the efficacy of the MLP scoring schema proposed by Barth et al. [13]. However, given that false Positive Rate was 5.7%, the MRI results must be complemented with other assessments to predict neurological outcomes in patients following cardiac arrest according to the current guidelines.

Two patients with favourable neurological outcomes (CPC 1 and 2) demonstrated MLP 4, in contrast EEG, NSE, and clinical examination including pupil- and corneal-reflexes did not predict UO. Both MRIs were conducted at 44 respectively 43 h after ROSC and notably, both patients were younger (30 and 45 years) than the median age of the analysed cohort. One explanation could be that these patients have had a reversible underlying overlapping pathology mimicking the classical image pattern of a severe hypoxic brain injury on MRI like for example a reversible cerebral vasoconstriction [17]. The imaging pattern in case of a coronavirus disease (COVID-19) or severe hypo-/hyperglycaemia can also have a similar appearance on MRI like global hypoxic injury patterns [17]. However, both patients were tested negative for COVID-19 and both were normoglycaemic during MRI episodes. That emphasize the importance of interpreting the MR imaging findings within the context of clinical examination and laboratory results. Additionally, in the initial study, the MLP score, serving as an anatomical description of lesions, was compared to EEG results, a functional examination of the brain. Both anatomical as well as functional examination don’t necessarily correlate with functional outcome. However, we believe that functional outcome represents the more important comparison, as this is more relevant for patients, families, and care team.

Early MRI for neuroprognostication has the great advantage of being independent of sedation, in contrast to clinical examination and EEG, where sedation is a well-described confounder. This makes MRI a valuable tool for neuroprognostication, especially if reduction of sedation is not tolerated by the patient. However, a consensus on the standardized quantification of the extent of brain injury in MRI is lacking, and there is also a deficiency in standardized protocols for MRI sequences.

Nevertheless, the use of MRI for neurological prognostication is on the rise, not only for patients after cardiac arrest but also for other unconscious patients in the ICU [18]. Typically, the DWI sequence between 48 and 72 h is considered as best prediction exam. This MRI sequence measures the diffusion of water molecules and illustrates the extent of cytotoxic brain oedema. Various classifications have been proposed, including the extension of cortical diffusion restriction [19], lesion topography cortical vs. subcortical [13], and the average ADC computed over the whole brain [20]. The utilization of artificial intelligence for analysis is an expanding field and might help grading the extent of hypoxic ischemic encephalopathy.

To reduce the risk of inaccurately predicting UO, which could lead to a ‘self-fulfilling prophecy’ and subsequent death after life support measures are withdrawn, it is recommended to conduct multimodal assessments and adopt a cautious approach in interpreting test results [8]. This strategy leads to a moderate sensitivity in neurological prognostication. In fact, only a minority of patients undergoing neuroprognostication are classified as having UO. M. Moseby-Knappe assessed the 2015 ERC/ESICM algorithm in patients from the TTM Trial [21]. Out of the 585 patients who underwent neuroprognostication, 103 patients (18%) were identified as having UO, while 163 patients (28%) did not meet the criteria but were found to have UO. This leaves a substantial number of patients and relatives with uncertainty. While the 5.7% false positive rate in our group prevents precise early prognosis, the negative likelihood ratio of 0.07 significantly increases the probability of accurate prognostication of FO. In regions where advance care directives with limitations of ICU treatment due to perceived futility are common, early MRI findings can provide caregivers and family members with valuable information about the likelihood of FO.

Our study has certain limitations. Despite the data being collected prospectively, it is a single-center retrospective analysis with a small patient sample size (n = 48 patients). The percentage of patients with FO is lower compared to contemporary trials like TTM2 and TAME [22, 23]. However, this reflects real-world data where all patients suffering from OHCA are included without bias in selection. This is particularly evident in the significant number of patients who experience early mortality due to circulatory failure and brain death as well as the number of patients who regained consciousness after 24 h and did not undergo neuroprognostication. Another limitation of this study is that treating clinicians were aware of the MRI results, which could introduce a potential self-fulfilling prophecy. However, in only 14% (n = 5) of the patients where care was withdrawn after UO was predicted, MRI was one of two modality indicating UO. This means 86% of these patients fulfilled the criteria for UO even without MRI, mitigating the bias of a self-fulfilling prophecy to some extent. Additionally, radiologists were blinded to the patients' outcomes.

Lastly, the high prevalence of patients with ultimately UO (75%) in our cohort may overestimate sensitivity and underestimate specificity. However, likelihood ratios are independent of prevalence and our results (Positive likelihood ratio > 5, Negative likelihood ratio < 0.1) demonstrate good to excellent probabilities for predicting of UO and FO.

The role of MRI in neuroprognostication is evolving. Current studies involve small patient cohorts, and there is no standardization in MRI timing, measurements, and post-processing techniques. Given this context, our study needs to be reproduced and compared regarding the timing of MRI and the quantification of MRI lesions. To date, early MRI cannot replace multimodal prognostication, but might play an important role in prediction of likelihood of FO/UO early in the course of ICU treatment. The MLP classification in early MRI in patients with hypoxic ischemic encephalopathy has reasonable sensitivity and specificity, but needs further confirmation in larger multicentre cohorts.

## Conclusion

We demonstrated the effectiveness of the MLP scoring scheme in predicting neurological outcome in patients following cardiac arrest. However, to ensure a comprehensive neuroprognostication, MRI results need to be combined with other assessments. While neuroimaging is a promising objective tool for neurological prognostication, given the absence of sedation-related confounders—compared to EEG and clinical examination—the current lack of a validated scoring system necessitates further studies. Incorporating standardized MRI techniques and grading systems is crucial for advancing the reliability of neuroimaging for neurological prognostication.

## Data Availability

The datasets used and/or analysed during the current study are available from the corresponding author on reasonable request.

## Abbreviations

ADC: Average apparent diffusion coefficient
COVID-19: Coronavirus Disease 2019
CPC: Cerebral performance category
DWI: Diffusion restriction sequence
ECMO: Extracorporal membrane oxygenation
EEG: Electroencephalogram
FO: Favourable outcome
ICU: Intensive Care Unit
IQR: Interquartile range
MLP: MR-lesion patterns
NSE: Neuron-specific enolase
OHCA: Out-of-hospital cardiac arrest
PTCA: Percutaneous transluminal coronary intervention
ROSC: Return of spontaneous circulation
SSEP: Somatosensory evoked potential
UO: Unfavourable outcome
